# Immune characterization of breast cancer metastases: prognostic implications

**DOI:** 10.1186/s13058-018-1003-1

**Published:** 2018-06-22

**Authors:** Maria Vittoria Dieci, Vassilena Tsvetkova, Enrico Orvieto, Federico Piacentini, Guido Ficarra, Gaia Griguolo, Federica Miglietta, Tommaso Giarratano, Claudia Omarini, Serena Bonaguro, Rocco Cappellesso, Camillo Aliberti, Grazia Vernaci, Carlo Alberto Giorgi, Giovanni Faggioni, Giulia Tasca, Pierfranco Conte, Valentina Guarneri

**Affiliations:** 1 Department of Surgery, Oncology and Gastroenterology, University of Padova, Via Gattamelata 64, 35128 Padova, Italy; 2 Medical Oncology 2, Istituto Oncologico Veneto IRCCS, Via Gattamelata 64, 35128 Padova, Italy; 3 Department of Pathology, Azienda Ospedaliera di Padova, Padova, Italy; 4 Department of Medical and Surgical Sciences of Mother, Child and Adult, University of Modena and Reggio Emilia, Modena, Italy; 5 Division of Pathology, University Hospital of Modena, Modena, Italy; 6 Department of Medical Oncology, University Hospital of Modena, Modena, Italy; 7 Surgical Pathology and Cytopathology Unit, Department of Medicine, University of Padova, Padova, Italy; 8 Radiology, Istituto Oncologico Veneto IRCCS, Padova, Italy

**Keywords:** Metastatic breast cancer, Tumor-infiltrating lymphocytes, PD-L1, Triple negative, HER2

## Abstract

**Background:**

Tumor-infiltrating lymphocytes (TILs) evaluated in primary breast cancer (BC) convey prognostic information. Limited data in the metastatic setting are available.

**Methods:**

Secondary lesions from 94 BC patients, 43 triple-negative (TN) and 51 HER2-positive, were evaluated for TILs and expression of CD8, FOXP3, and PD-L1 by immunohistochemistry.

**Results:**

TILs levels on metastasis were generally low (median 5%) and did not differ between TN and HER2+ tumors. Younger patients showed significantly lower TILs (*p* = 0.002). In HER2+ patients, TILs were higher in lung metastases as compared to other sites (*p* = 0.038). TILs composition was different across metastatic sites: skin metastases presented higher FOXP3 (*p* = 0.002) and lower CD8/FOXP3 ratio (*p* = 0.032). Patients treated for metastatic BC prior to biopsy had lower CD8 (overall: *p* = 0.005, HER2+: *p* = 0.011, TN: *p* = 0.075).

In TN patients, median overall survival (OS) was 11.8 and 62.9 months for patients with low and high TILs, respectively (HR 0.29, 95%CI 0.11–0.76, log-rank *p* = 0.008). CD8/FOXP3 ratio was also prognostic in TN patients (median OS 8.0, 13.2, and 54.0 months in 1st, 2nd and 3th tertile, log-rank *p* = 0.019). Both TILs and CD8/FOXP3 ratio were independent factors at multivariate analysis. Counterintuitively, in HER2+ BC, low TILs tumors showed better prognosis (median OS 53.7 vs 39.9 months in TILs low and TILs high, not statistically significant).

**Conclusions:**

Our findings indicate the relevance of TILs as prognostic biomarker for TNBC even in the advanced setting and provide novel hypothesis-generating data on potential sources of immune heterogeneity of metastatic BC.

**Electronic supplementary material:**

The online version of this article (10.1186/s13058-018-1003-1) contains supplementary material, which is available to authorized users.

## Background

Tumor-infiltrating lymphocytes (TILs) are believed to reflect the immunogenicity of breast cancer (BC), with rapidly accumulating evidence suggesting the clinical validity and potential utility of TILs as a biomarker [1]. Most of available data focus on early BC, where TILs have proved to retain a strong prognostic value especially in triple-negative (TN) and human epidermal growth factor receptor-2 (HER2) positive BC, when assessed on hematoxylin and eosin-stained (HES) sections of primary tumor in accordance with International Immuno-Oncology Biomarker Working Group recommendations [2–9]. In addition, an association between TILs and response to preoperative chemotherapy (plus anti-HER2 agents if HER2-positive) has been reported [10–15]_._

In contrast to the early setting, little is known on the prognostic role of TILs in advanced disease for patients treated with standard therapies currently available. In this context, prospective-retrospective data come from translational analyses of two trials evaluating combinations of chemotherapy and anti-HER2 agents as first-line for HER2-positive metastatic breast cancer patients [16, 17]. In the Cleopatra trial (trastuzumab+docetaxel vs pertuzumab+trastuzumab+docetaxel), longer OS was observed in patients with high TILs levels [16], whereas in the MA.31 trial (trastuzumab+taxane vs lapatinib+taxane) poorer prognosis was observed in case of low CD8-positive TILs on tumor samples from patients treated in the lapatinib arm relative to those receiving trastuzumab [17]. However, as a main limitation of these studies, TILs were assessed almost exclusively on primary tumor samples. Thus, it remains largely unexplored whether the evaluation of TILs in secondary lesions could provide more accurate information on the actual immunological balance between tumor and host. Indeed, available evidence suggests that immune microenvironment may differ between primary tumor and its paired secondary lesions in terms of both TILs levels and composition [18–21].

In the present study we aim to assess TILs levels, immune infiltrate composition and programmed death-ligand 1 (PD-L1) expression in metastatic lesions and to evaluate their prognostic impact for patients with TN and HER2+ advanced BC.

## Methods

### Study population

We retrospectively included 94 patients with HER2+ or TN (HR-/HER2-) metastatic BC diagnosed before 2015 at IRCCS Istituto Oncologico Veneto in Padua and Modena University Hospital for whom histologic material obtained with surgical resection or biopsy of regional or distant recurrence was available (lymphoid tissue metastasis were excluded).

Patients were identified from a prospectively maintained institutional database, in which clinicopathological characteristics (including histological type, tumor grade, hormonal receptor, HER2 status and clinical stage at diagnosis), time and site of recurrence and follow-up data were recorded.

Patients with ipsilateral in-breast recurrences were excluded given the difficulty in discriminating between a recurrence and a new primary lesion.

The study protocol was approved by local ethics committee.

### Pathology assessments

Formalin-fixed paraffin-embedded tumor samples from metastatic sites and, when available, matched primary tumor samples were retrieved.

Estrogen receptor (ER) and progesterone receptor (PgR) were considered positive if immunohistochemistry (IHC) staining is positive in ≥10% of tumor cells. HER2 was considered negative if it was present in IHC staining of 0/1+ and/or FISH non-amplified.

#### TILs evaluation

Hematoxylin and eosin-stained (HES) slides were retrieved from Institutional Pathology archives. Stromal TILs were assessed according to consensus guidelines [3, 22] by two independent investigators (MVD and FM), blinded for clinical data. The average value was used for analyses.

#### Immunohistochemistry

CD8 and FOXP3 IHC staining was performed by using the fully automated stainer BOND III (Leica, Wetzler, Germany). Paraffin slides were deparaffinized in xylene and rehydrated through graded alcohols. Antigen retrieval was performed with slides heated in EDTA buffer (pH 9.0) for 20 min at 100 °C.

After antigen retrieval, the slides were allowed to cool. The slides were rinsed with Tris-buffered saline (TBS) and the endogenous peroxidase was inactivated with 3% hydrogen peroxide block at room temperature. After protein block, the slides were incubated with primary antibody to human CD8 (Monoclonal Mouse Anti-Human CD8, Clone C8/144B, dilution 1:100, Dako Cytomation, Glostrup, Denmark) and FOXP3 (Monoclonal Mouse Anti-Human FOXP3, Clone 236A/E7, dilution 1:200, Abcam, Cambridge, MA, USA) for 15 min. The sections were rinsed in TBS and incubated for 8 min with the Post-Primary Antibody (Leica Biosystems). Then, the sections were rinsed in TBS and incubated for 8 min with Polymer (Leica Biosystems). Finally, peroxidase reactivity was visualized using a Mixed DAB Refine, then the sections were counterstained with hematoxylin for 15 min and mounted.

PD-L1 IHC staining was performed by using the Ventana automated immunostainer BenchMark ULTRA. (Ventana Medical Systems, Tucson, AZ, USA). The slides were dried at 60 °C for 1 h and deparaffinized using EZ Prep (Ventana Medical Systems) at 72 °C. Cell conditioning was performed using ULTRA CC1 solution (Ventana Medical Systems) at 100 °C for 64 min. Ventana PD-L1 Primary Antibody (rabbit monoclonal, clone SP263 ref. 790–4905, Ventana Medical Systems) was incubated at 36 °C for 16 min. Signals were detected using the OptiView DAB IHC Detection Kit (Ventana Medical Systems). Counterstaining was performed with Hematoxylin II and Bluing reagent (Ventana Medical Systems).

#### CD8, FOXP3 and PD-L1 scoring

Evaluation of CD8, FOXP3 and PD-L1 expression was performed by a pathologist, blinded for clinical data.

For CD8 and FOXP3, five fields of 2–3 mm of diameter were analyzed at ×40 for each sample. The five field were selected within the area identified for the evaluation of TILs according to available recommendations [3, 22] and were in the area exhibiting tumor invasion. The number of positive immune cells in the five selected fields was counted on a light microscope. For each sample, the average number of stained immune cells across the evaluated fields was calculated.

PD-L1 expression was assessed on the entire section. PD-L1 was evaluated on tumor cells (% of cells positively stained/total tumor cells) and on stromal/immune cells in tumor stroma (% of cells positively stained/total cells in tumor stroma).

PD-L1 expression was found to be predominant in stromal/immune cells rather than tumor cells, mean expression on tumor cells was 7%, mean expression on stromal/immune cells was 15%, *t* test *p* = 0.003. Expression in the two cells compartments was strongly and positively correlated (Spearman’s coefficient 0.554, *p* < 0.001). Therefore, we decided to consider stromal/immune cells expression for further analyses in this study.

### Statistical analysis

Statistical analysis was carried out using IBM SPSS (version 24) software (IBM Corp, Armonk, NY, USA).

Descriptive statistics were performed for patient demographics and clinical characteristics. For continuous variables median, range values and quartiles were computed. The Mann-Whitney nonparametric test was used to study the distribution of continuous variables across groups defined by clinicopathologic characteristics.

The Spearman’s rank correlation coefficient was used to study the correlation between continuous variables.

Overall survival (OS) was defined as the time from first relapse to death from any cause. Alive patients were censored at the date of last follow-up. Median OS was estimated using the Kaplan–Meier method and reported with 95% confidence intervals (95% CIs). The Kaplan–Meier method was used to estimate survival curves, the log-rank test was used to compare between groups. Univariate and multivariate Cox regression modeling for proportional hazards was used to calculate HR and 95% CI. All reported *p* values are two-sided, and significance level was set at *p* < 0.05.

The association between TILs levels as continuous variable and OS was tested. The prognostic value of TILs levels categorized using a 10% cutoff [16, 20, 23] was also evaluated. The prognostic value of CD8/FOXP3 ratio was tested using tertiles.

We tested two distinct cutoffs for PD-L1 expression in stromal/immune cells (1% and 5%) and its association with OS.

## Results

### Clinicopathologic characteristics and association with TILs levels

In the present study, tumor samples of BC recurrences obtained from 94 patients were assessed (*n* = 43 TN and *n* = 51 HER2+) for TILs.

Patients’ baseline characteristics are reported in Table 1. Median time from relapse to sample collection was 0 months, the majority of patients (*n* = 70, 74%) underwent biopsy of recurrent BC before starting any systemic treatment for advanced disease. Most of the patients received prior neoadjuvant systemic treatment (86% of patients received chemotherapy, 59% of HER2+ patients received trastuzumab). The majority of tumor samples were obtained from distant relapses (57%).

**Table 1:** Patients’ baseline characteristics: overall and according to tumor subtype

	Population evaluated for TILs levels			Population evaluated for CD8, FOXP3, PD-L1		
	Overall n (%)	TN n (%)	HER2+ n (%)	Overall n (%)	TN n (%)	HER2+ n (%)
Tot, n	94	43	51	64	28	36
Age at BC diagnosis, yrs. median (Q1–Q3)	49 (42–57)	51 (44–61)	46 (39–53)	51 (43–59)	54 (45–63)	47 (41–57)
Time to relapse, mos median (Q1–Q3)	27 (15–43)	19 (11–38)	31 (19–60)	27 (16–47)	18 (11–34)	31 (21–55)
Time from relapse to biopsy, mos median (Q1–Q3)	0 (0–5)	0 (0–2)	0 (0–11)	0 (0–6)	0 (0–2)	0 (0–15)
Histotype						
Ductal	81 (86)	36 (84)	43 (84)	56 (88)	22 (79)	34 (94)
Lobular	5 (5)	1 (2)	4 (8)	2 (3)	1 (4)	1 (3)
Other	8 (9)	6 (14)	2 (4)	6 (9)	5 (17)	1 (3)
Grade						
1–2	13 (14)	3 (7)	10 (20)	9 (14)	2 (7)	7 (19)
3	78 (86%)	39 (93)	39 (80)	54 (86)	25 (83)	29 (81)
HR status						
HR+	37 (39)	0 (0)	37 (72)	26 (41)	0 (0)	26 (72)
HR-	57 (61%)	43 (100)	14 (28)	38 (59)	28 (100)	10 (28)
Stage at diagnosis						
I–II	50 (55)	27 (66	23 (46)	33 (52)	17 (63)	16 (44)
III	38 (42)	13 (32)	25 (50)	27 (43)	9 (33)	18 (50)
IV	3 (3)	1 (2)	2 (4)	3 (5)	1 (4)	2 (6)
Type of recurrent BC sample						
Locoregional relapse	40 (43)	23 (53)	17 (33)	25 (39)	13 (46)	12 (33)
Distant relapse	54 (57)	20 (47)	34 (67)	39 (61)	15 (54)	24 (67)
Organ/site of biopsy						
Liver	24 (25)	5 (12)	19 (37)	15 (23)	4 (14)	11 (31)
Skin	38 (40)	21 (49)	17 (33)	26 (41)	13 (47)	13 (36)
Lung	12 (13)	7 (16)	5 (10)	8 (13)	4 (14)	4 (11)
CNS	9 (10)	4 (9)	5 (10)	9 (14)	4 (14)	5 (14)
Other	11 (12)	6 (14)	5 (10)	6 (9)	3 (11)	3 (8)
Neo/adjuvant chemotherapy						
No	13 (14)	3 (7)	10 (20)	9 (14)	3 (11)	6 (17)
Yes	81 (86)	40 (93)	41 (80)	55 (86)	25 (89)	30 (83)
Neo/adjuvant trastuzumab						
No	–	–	21 (41)	–	–	12 (33)
Yes	–	–	30 (59)	–	–	24 (67)
Pre-biopsy systemic treatment for MBC						
No	70 (74)	36 (88)	34 (67)	46 (72)	25 (89)	21 (58)
Yes	24 (26)	7 (17)	17 (33)	18 (28)	3 (11)	15 (42)

*Abbreviations*: *TILs* tumor-infiltrating lymphocytes, *TN* triple negative, *HER2* human epidermal growth factor receptor-2, *n* number, *tot* total, *BC* breast cancer, *yrs* years, *mos* months, *Q1* first quartile, *Q3* third quartile, *HR* hormone receptor, *CNS* central nervous system, *MBC* metastatic breast cancer

Median TIL levels in the overall population was 5% (Q1 2%; Q3 10%), with similar results in TN and HER2+ patients (median 5%, Q1 2.25% to Q3 10% for TN and median 5%, Q1 2% to Q3 11% for HER2+; *p* = 0.885), as shown in Table 2.

**Table 2:** TILs distribution according to tumor subtype and clinical characteristics

	TILs %, median (Q1 – Q3): all patients 5 (2–10)					
	Overall	*p* value	TN	*p* value	HER2+	*p* value
Tumor phenotype						
TN	5 (2–10)		–		–	
HER2+	5 (2–11)	0.855	–	–	–	–
Age at BC diagnosis						
≤ 50 years	3 (2–8)	**0.002**	3 (2–10)	**0.037**	4 (2–8)	**0.010**
> 50 years	8 (5–15)		7 (4–11)		9 (5–18)	
HR status						
Negative	–	–	–	–	9 (5–15)	**0.029**
Positive	–		–		4 (2–8)	
Organ/site of biopsy						
Liver	5 (2–8)	0.277	5 (5–11)	0.936	5 (2–8)	0.119
Skin	3(2–10)		3 (2–10)		4 (2–10)	
Lung	9 (5–18)		8 (3–10)		18 (6–19)	
CNS	6 (3–11)		4 (3–38)		11 (5–11)	
Other	5 (2–8)		5 (2–19)		5 (2–8)	
Pre-biopsy systemic treatment for MBC						
No	5 (3–11)	0.563	5 (3–10)	0.104	4 (2–13)	0.652
Yes	5 (2–8)		2 (2–5)		6 (5–9)	

*Abbreviations*: *TILs* tumor-infiltrating lymphocytes, *Q1* first quartile, *Q3* third quartile, *p p* value, significant values in bold, *TN* triple negative, *HER2* human epidermal growth factor receptor-2, *BC* breast cancer, *HR* hormone receptor, *CNS* central nervous system, *MBC* metastatic breast cancer

Although TILs were not significantly different across biopsy sites, lung samples showed the highest levels among all sites. The comparison of TILs in lung versus other metastatic sites (all together) was of borderline significance in the overall cohort (*p* = 0.084) and reached statistical significance in the HER2+ cohort (*p* = 0.038). In the entire cohort, skin samples showed the lowest median TILs levels among all metastatic sites (3.5%), with similar results in the TN and HER2+ cohorts separately. However, when comparing skin versus other metastatic sites altogether, results were not statistically significant (*p* = 0.312 in the entire cohort, *p* = 0.418 in the TN cohort and *p* = 0.548 in the HER2+ cohort).

Women who were younger at the time of BC diagnosis (≤50 years) showed significantly lower TILs on metastasis than older patients (> 50 years) both in the overall population (*p* = 0.002) and when each tumor subtype was considered separately (*p* = 0.037 and *p* = 0.010 and for TN and HER2+ patients, respectively).

In the HER2+ population, TILs were significantly lower in case of HR+ disease (*p* = 0.029).

In the TN cohort, there was a nonsignificant trend for lower TILs for patients who received chemotherapy for metastatic disease prior to metastasis biopsy, as compared to patients undergoing biopsy before starting first-line treatment (median 2%, Q1 2% to Q3 5%; median 5%, Q1 23% to Q3 10%, respectively; *p* = 0.104).

Matched primary tumors were available for only 55 patients. TILs were not significantly different in primary versus matched metastasis (median 5%, Q1 2.5% to Q3 12.5% and median 6.25, Q1 2% to Q3 12.5%, *p* = 0.925). In the TN cohort (*n* = 27), TILs tended to decrease in metastasis, although not significantly (median 7.5%, Q1 2.5% to Q3 20% in primary and median 6.25, Q1 2% to Q3 11% in metastasis, *p* = 0.477). In HER2+ patients TILs were nonsignificantly higher in metastases (median 2.5%, Q1 2% to Q3 7% in primary and median 6%, Q1 2% to Q3 14% in metastasis, *p* = 0.452).

### TILs composition

CD8 and FOXP3 were assessed on 64 biopsies (*n* = 36 HER2+, *n* = 28 TN), while PD-L1 expression was available for 62 cases (*n* = 35 HER2+, n = 27 TN). Patients’ baseline characteristics are reported in Table 1.

**Fig. 1:**
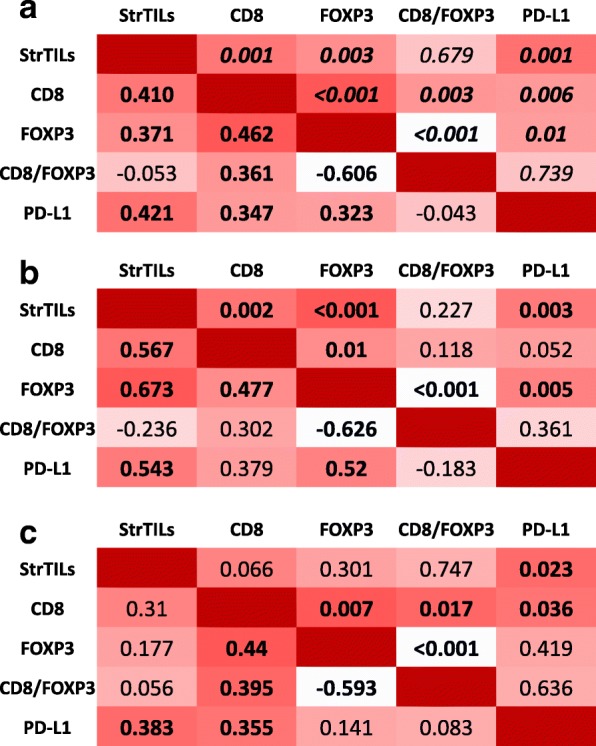
Correlations between immune parameters. Heatmap showing Spearman’s coefficients and statistical significance (*p* values) for the correlation between immune markers in the overall population (**a**), TN cohort (**b**) and HER2+ cohort (**c**). Significant *p* values in bold. *PD-*L1 programmed death-ligand 1, *TILs* tumor-infiltrating lymphocytes

Figure 1 shows a heatmap with Spearman’s coefficients and *p* values for the correlation between immune markers. In the entire cohort, CD8, FOXP3, PD-L1, and TILs showed a significant positive correlation with each other. The Spearman’s coefficients showed generally a moderate correlation between the variables, with the exception of weak correlations between TILs and FOXP3, PD-L1 and CD8, and PD-L1 and FOXP3.

The highest Spearman’s correlation coefficients between TILs, CD8, FOXP3, and PD-L1 were observed in the TN cohort. The correlations were mostly moderate, with the exception of a strong correlation between TILs and FOXP3 and a weak correlation between CD8 and PD-L1.

In the HER2 cohort, correlation coefficients between TILs, CD8, FOXP3, and PD-L1 were the lowest. Correlations were very weak to weak, with the exception of the one between CD8 and FOXP3 that showed a Spearman’s coefficient of 0.440.

CD8/FOXP3 ratio was obviously positively and negatively correlated with CD8 and FOXP3, respectively, and did not correlate with TILs or PD-L1.

Full data regarding distribution of CD8, FOXP3, CD8/FOXP3 ratio, and PD-L1 according to tumor subtype and other clinical variables is detailed in (Additional file 1: Tables S1; Additional file 2: Table S2; Additional file 3: Table S3; and Additional file 4: Table S4)**.**

Distribution of these parameters did not vary significantly according to tumor subtype.

**Fig. 2:**
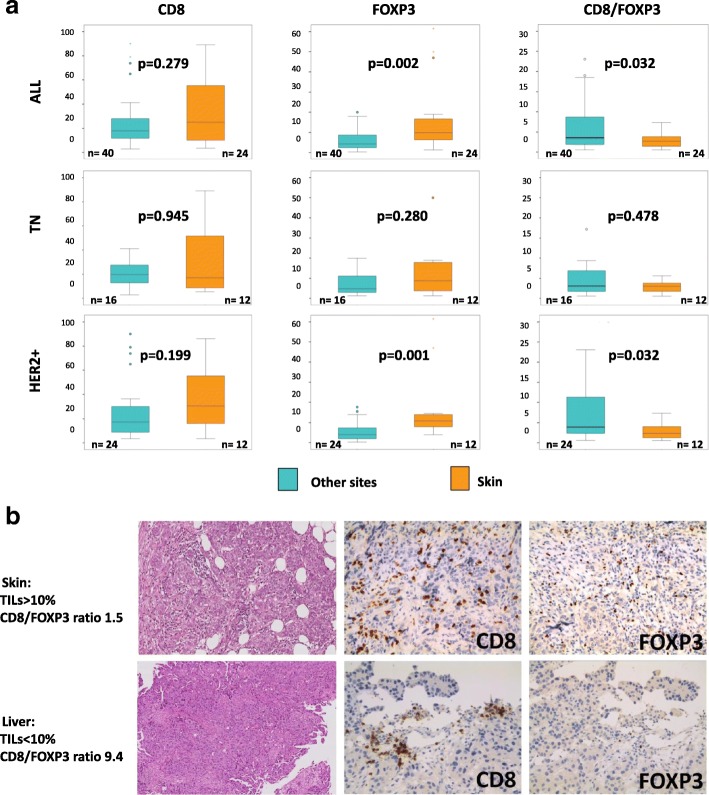
CD8, FOXP3, and CD8/FOXP3 ratio in skin versus other metastasis sites. Boxplots comparing CD8, FOXP3, and CD8/FOXP3 ratio in skin versus other metastasis sites in all patients and in TN and HER2+ cohorts separately (**a**). Pictures showing a skin metastasis with high TILs and low CD8/FOXP3 ratio and a liver metastasis with low TILs and high CD8/FOXP3 ratio (**b**). *HER2* human epidermal growth factor receptor-2, *TILs* tumor-infiltrating lymphocytes, *TN* triple negative.

Levels of FOXP3 were significantly different across site of biopsy (0.046). In particular, FOXP3 was significantly higher in skin metastases as compared to other metastatic sites considered all together (*p* = 0.002). Accordingly, CD8/FOXP3 ratio was the lowest in skin biopsies as compared to other metastatic sites (*p* = 0.032). This trend was seen both in the HER2+ and TN subgroup, though it only reached statistical significance in the HER2+ subgroup, as shown in Fig. 2.

Patients who underwent biopsy of metastatic lesion prior to start first-line treatment showed significantly higher CD8+ infiltrating lymphocytes as compared to patients who previously received systemic treatment for advanced disease: *p* = 0.005 for overall population and *p* = 0.011 for HER2+ tumors. The analysis in the TN cohort (*p* = 0.075) was limited by the fact that only three patients underwent metastasis biopsy prior to first-line therapy. Most of the HER2+ patients who received systemic therapy for metastatic disease prior to biopsy received both chemotherapy and anti-HER2 (13/17, 76.5%).

CD8/FOXP3 ratio was higher in HR+/HER2+ as compared to HR-/HER2+ patients (*p* = 0.052); however, this might in part be confounded by the fact that more HR-/HER2+ patients had skin metastasis biopsies than HR+/HER2+ patients (43% vs 27%, *p* = 0.277).

### Impact on survival

Due to profound differences in disease course and therapeutic options, the prognostic value of immune biomarkers was assessed separately in the TN and HER2+ cohorts.

**Fig. 3:**
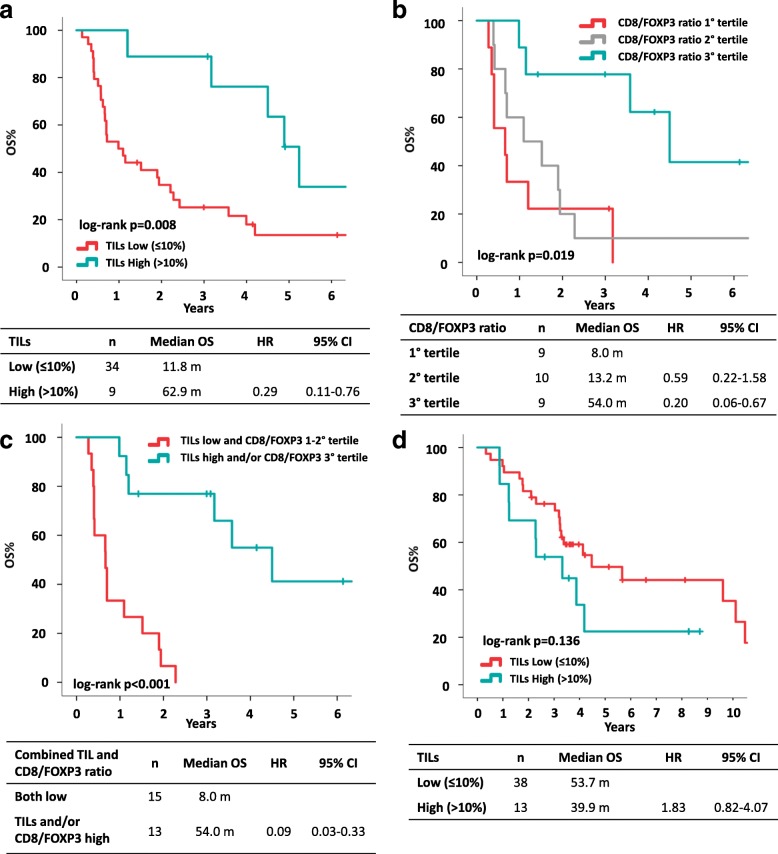
Overall survival (OS) according to immune biomarkers assessed on metastasis. OS in the triple-negative cohort according to TILs (**a**), CD8/FOXP3 ratio (**b**) and CD8/FOXP3 ratio combined with TILs (**c**); OS in the HER2+ cohort according to TILs levels (**d**). *CI* confidence interval, *HR* hazard ratio, *OS* overall survival, *TILs* tumor-infiltrating lymphocytes

In the TN population, TILs had a significant prognostic impact on OS (median OS 11.8 months vs 62.9 months for TILs low and TILs high, respectively; HR 0.29, 95% CI 0.11–0.76, *p* = 0.012) (Fig. 3a). Each 1% increase in TILs was associated with a 3% reduction in the risk of death (HR 0.97, 95% CI 0.94–1.00, *p* = 0.075). Since we did not show significantly different TILs levels between primary and metastasis in the 27 TN cases with available samples, we explored, in this subgroup, the prognostic value of TILs in primary tumor and in metastasis. TILs in metastasis were significantly associated with OS (HR 0.29, 95% CI 0.09–0.91, *p* = 0.033), whereas TILs in primary tumor did not (HR 0.50, 95% CI 0.19–1.34, *p* = 0.170). In addition, CD8/FOXP3 ratio on metastasis also showed a prognostic role in TN patients (median OS 8.0, 13.2 and 54.0 months in the first, second and third tertile, respectively; *p* = 0.019) (Fig. 3b). TILs and CD8/FOXP3 ratio were weakly correlated (Fig. 1), indeed, biopsies with high CD8/FOXP3 ratios (third tertile) were almost exclusively characterized by low TILs (eight out of nine cases with high CD8/FOXP3 ratio). In a multivariate Cox regression analysis both TILs and CD8/FOXP3 ratio maintained an independent prognostic value in TN tumors (Table 3) and the combined use of TILs and CD8/FOXP3 ratio allowed refining the prognostic ability of single biomarkers (median OS 8.0 months for tumors with low TILs and low CD8/FOXP3 ratio vs 54.0 months for tumors with high TILs and/or high CD8/FOXP3 ratio, *p* < 0.001, Fig. 3c).

**Table 3:** OS univariate and multivariate analysis of immune parameters evaluated on metastasis biopsies of triple-negative breast cancer

			Univariate		Multivariate
		Median OSmonths (95%CI)	HR (95%CI)	*p*	HR (95%CI)
TILs	Low ≤10%	11.8 (4.1–19.5)	ref	**0.012**	ref
	High > 10%	62.9 (52.3–73.5)	0.29 (0.11–0.76)		0.06 (0.01–0.29)
CD8/FOXP3 ratio	1st tertile	8.0 (0.0–17.1)	ref	**0.020**	ref
	2nd tertile	13.2 (0.0–28.3)	0.59 (0.22–1.58)		0.17 (0.05–0.59)
	3rd tertile	54.0 (32.4–75.7)	0.20 (0.06–0.67)		0.03 (0.004–0.15)

*Abbreviations*: *OS* overall survival, *HR* hazard ratio, *p p* value, *TILs* tumor-infiltrating lymphocytes

Significant *p* values in bold

In the HER2+ population, an inverse relationship between TILs and prognosis was observed, with patients with lower TILs showing better prognosis (not statistically significant): median OS was 53.7 months vs 39.9 months for TILs low and TILs high, respectively; *p* = 0.136 (Fig. 3d). Results were similar in HR-/HER2+ and HR+/HER2+ patients, although the analysis was limited by reduced sample size. In HR-/HER2+, median OS was 50.3 months vs 15.1 months for patients with low and high TILs, respectively (HR 2.45, 95%CI 0.54–11.16). In HR+/HER2+ patients median OS was 68.9 and 47.1 months in case of low and high TILs, respectively (HR 1.54, 95% CI 0.55–4.28). CD8/FOXP3 ratio did not show any prognostic role (Additional file 5: Figure S1).

PD-L1 expression in tumor stroma was not prognostic in TN and in HER2+ tumors when considering a 5% cutoff (Additional file 6: Figure S2). Similar results were obtained with a 1% cutoff (data not shown).

## Discussion

This article reports the assessment of immune-related biomarkers for patients with TN and HER2+ metastatic breast cancer. The series of samples analyzed in this work represents the largest published series of metastatic breast cancer samples analyzed for immune biomarkers. In our study, TILs were generally low. It is now recognized that patients with HER2+ and TN early BC with high levels of TILs on primary tumor have a lower recurrence rate [2–9], therefore suggesting that BC recurrences might be enriched in low TILs tumors. Moreover, previous reports showed lower TIL levels in secondary lesions as compared to primary tumors [16, 18, 19]. We only found a nonsignificant decrease in TILs from primary to metastasis in the TN cohort of our study, however, the sample size of patients with matched samples was small.

We observed that host-dependent factors are associated with TILs on metastasis. In particular, younger women showed lower levels of TILs than older patients. This is the first time that such correlation is reported for patients with metastatic disease. The reason for lower TILs levels in younger patients is largely unknown. A potential underexplored hypothesis is that younger patients present higher levels of circulating estrogens, which might have an immunosuppressive action [24, 25]. However, most of the patients had received neoadjuvant chemotherapy, which might have induced iatrogenic menopause and, unfortunately, it was not possible to capture the menopausal status of these patients at the time of metastatic disease onset. Other host-related factors such as race have been also shown to be associated with differing levels of immune infiltrate in a previous study [16].

We found in our analysis that the characteristics of the tumor immune infiltrate may be different across metastatic sites. We observed the highest TILs levels in lung metastasis and the lowest levels in skin secondary lesions, which is consistent with previous data [16, 20]. Interestingly, we also could appreciate differences in immune infiltrate composition. In particular, skin metastasis presented significantly higher FOXP3 levels and lower CD8/FOXP3 ratio, as compared to other biopsy sites. This may suggest that cutaneous tissue might harbor a more permissive immune microenvironment for tumor growth. A physiological mitigation of the cytotoxic immune activity in skin tissue through different immunosuppressive mechanisms, a process known as “immune privilege”, has been described by several authors [26, 27]. The immune heterogeneity across metastatic sites deserves to be further explored, due to potential relevance in explaining heterogeneous response to standard treatments and to immunotherapy.

Another factor that was found to be significantly related to TILs composition is previous treatment. Having received CT or anti-HER2 therapy in the metastatic setting prior to biopsy was associated with lower CD8+ TILs. Recently, Loi et al. reported lower levels of TILs for TN patients treated with multiple lines of chemotherapy as compared to patients who did not receive treatment for metastatic disease [20]. These data suggest that heavily pretreated patients might have an impaired antitumor cytotoxic activity of the immune system thus possibly explaining, at least in part, data from immune checkpoint inhibitor trials showing better response rates in first-line treatment than in subsequent lines [28–30].

TILs showed a strong prognostic value in TN BC, further corroborating the relevance of TILs as a biomarker in this disease subtype across different settings. TILs were significantly correlated with CD8, FOXP3, and PD-L1 in TN disease in our study, suggesting that the levels of TILs in TN reflect a general activation of the immune system. This observation supports TILs as a simple method to appreciate the immune activation status of a TN tumor. Indeed, given the availability of a standardized methodology for TILs assessment in the metastatic setting, the evaluation of this immune marker is technically simple and clinically reliable [22]. In addition, CD8/FOXP3 ratio was capable of identifying an additional subgroup of TN BC patients at good prognosis, which were not captured by the high TILs group. This hypothesis-generating result highlights that a finer evaluation of tumor microenvironment may be worthy of investigation in future studies.

Other well-known immune-related markers, such as PD-L1 expression, did not significantly affect outcome.

The clinical utility of TILs may not be confined to its prognostic value; in fact in recent studies of immune check-point inhibitors for metastatic TN BC patients, TILs rather than PD-L1 are a better predictor of response to immunotherapy [20, 23].

A different scenario was observed in HER2+ BC, where we did not observe any favorable impact of high TILs on OS. This result may appear in conflict with data from Cleopatra study [16], where higher TIL values were associated with improved OS. However, our HER2+ population was mostly represented by patients previously treated with anti-HER2 agents (60%) with samples obtained from metastases, while in the Cleopatra trial only a small proportion of patients was pretreated with anti-HER2 therapy (10.9%) and the majority of samples analyzed came from the primary tumor. As previously discussed, pretreatment with CT or HER2-targeted therapy may modify tumor immune microenvironment leading to impaired antitumor cytotoxic activity of the immune system.

## Conclusions

The main results of this study further stress the relevance of TILs as a prognostic biomarker for TN even in the advanced setting and provide novel hypothesis-generating data with regards to immune composition across different metastatic sites and the influence of previous treatments. Finally, the more complex interplay between the immune system and HER2+ BC deserve further exploration in the metastatic setting.

## Additional files

Additional file 1: **Table S1.** CD8 levels distribution according to tumor subtype and clinicopathological features. (DOCX 18 kb)

Additional file 2: **Table S2.** FOXP3 levels distribution according to tumor subtype and clinicopathological features. (DOCX 18 kb)

Additional file 3: **Table S3.** CD8/FOXP3 ratio distribution according to tumor subtype and clinicopathological features. (DOCX 18 kb)

Additional file 4: **Table S4.** PD-L1 levels distribution according to tumor subtype and clinicopathological features (DOCX 17 kb)

Additional file 5: **Figure S1.** Overall survival for HER2+ patients according to CD8/FOXP3 ratio. (PPTX 80 kb)

Additional file 6: **Figure S2.** Overall survival according to PD-L1 expression in HER2+ patients (2A) and TN patients (2B). (PPTX 91 kb)
