# Supplementary material for: Immune characterization of breast cancer metastases: prognostic implications
Source: Breast Cancer Res. 2018 Jun 22;20:62. doi: 10.1186/s13058-018-1003-1 (PMC6013851; doi:10.1186/s13058-018-1003-1)
Supplement: Supplementary file 1 — Table S1. CD8 levels distribution according to tumor subtype and clinicopathological features. (DOCX 18 kb) [file 13058_2018_1003_MOESM1_ESM.docx]

**Table S1. CD8 levels distribution according to tumor subtype and clinicopathological features.**

|  | **CD8 median (Q1-Q3): all patients 18.80 (11.30-35.50)** | | | | | |
| --- | --- | --- | --- | --- | --- | --- |
|  | **Overall** | ***P*** | **TN cohort** | ***P*** | **HER2+ cohort** | ***P*** |
| **Tumor phenotype**  **TN**  **HER2+** | 17.55 (11.68-32.55)  19.00 (10.00-41.80) | 0.766 | -  - | - | -  - | - |
| **Age at BC diagnosis**  **≤50 years**  **>50 years** | 19.00 (11.75-33.00) 16.60 (10.75-41.20) | 0.888 | 17.55 (11.75-23.80)  19.40 (10.75-33.60) | 0.981 | 19.50 (15.20-33.00)  15.67 (8.60-65.00) | 0.950 |
| **HR status**  **Negative**  **Positive** | -  - | - | -  - | - | 21.68 (8.20-53.33)  19.00 (11.00-36.20) | 0.903 |
| **Site of biopsy**  **liver**  **skin**  **lung**  **CNS**  **Other** | 17.60 (9.00-36.20)  23.10 (10.75-53.33)  17.75 (13.83-25.25)  22.00 (8.20-23.80)  18.80 (11.30-35.50) | 0.492 | 24.30 (10.30-36.10)  17.50 (9.33-42.20)  17.50 (9.33-42.20)  22.73 (19.30-23.53)  13.67 (11.75-33.60) | 0.975 | 16.50 (9.00-36.20)  28.00 (19.50-53.33)  19.00 (17.33-46.40)  8.20 (5.00-24.75)  15.00 (7.67-65.00) | 0.266 |
| **Prebiopsy systemic treatment for MBC**  **No**  **Yes** | 23.53 (15.20-42.20) 11.50 (8.20-18.60) | 0.005 | 22.20 (13.67-33.60)  10.75 (8.00-12.00) | 0.075 | 28.00 (15.67-57.40)  15.00 (8.20-19.00) | 0.011 |

Abbreviations: Q1, first quartile; Q3, third quartile; p, p value; TN, triple negative, BC, breast cancer, HR, hormone receptors; CNS, central nervous system, MBC, metastatic breast cancer
