# Supplementary material for: Immune characterization of breast cancer metastases: prognostic implications
Source: Breast Cancer Res. 2018 Jun 22;20:62. doi: 10.1186/s13058-018-1003-1 (PMC6013851; doi:10.1186/s13058-018-1003-1)
Supplement: Supplementary file 2 — Table S2. FOXP3 levels distribution according to tumor subtype and clinicopathological features. (DOCX 18 kb) [file 13058_2018_1003_MOESM2_ESM.docx]

**Table S2. FOXP3 levels distribution according to tumor subtype and clinicopathological features.**

|  | **FOXP3 median (Q1-Q3): all patients 6.43 (2.93-12.60)** | | | | | |
| --- | --- | --- | --- | --- | --- | --- |
|  | **Overall** | ***P*** | **TN cohort** | ***P*** | **HER2+ cohort** | ***P*** |
| **Tumor phenotype**  **TN**  **HER2+** | 6.79 (3.03-14.60)  6.40 (2.90-12.00) | 0.421 | -  - | - | -  - | - |
| **Age at BC diagnosis**  **≤50 years**  **>50 years** | 6.25 (2.67-12.00)  8.00 (3.20-13.40) | 0.436 | 4.33 (2.60-13.40)  8.68 (4.00-18.00) | 0.208 | 6.60 (4.00-9.50)  4.33 (2.00-13.40) | 0.849 |
| **HR status**  **Negative**  **Positive** | -  - | - | -  - | - | 8.54 (6.25-14.50)  4.33 (2.00-9.50) | 0.126 |
| **Site of biopsy**  **liver**  **skin**  **lung**  **CNS**  **Other** | 4.33 (2.40-8.00)  9.42 (6.25-15.80)  3.70 (2.10-10.50)  4.40 (2.60-7.75)  3.33 (2.00-13.20) | 0.046 | 8.45 (2.95-15.70)  8.75 (4.80-17.50)  6.50 (3.70-14.50)  4.83 (3.50-6.93)  2.33 (1.25-13.20) | 0.641 | 4.33 (2.00-7.00)  9.50 (7.25-13.40)  2.10 (0.67-7.60)  4.00 (2.60-7.75)  4.33 (2.00-15.50) | 0.050 |
| **Prebiopsy systemic treatment for MBC**  **No**  **Yes** | 6.75 (3.50-12.00)  6.43 (2.00-14.00) | 0.550 | 6.25 (3.40-13.20)  19.00 (2.50-20.00) | 0.314 | 7.25 (4.00-12.00)  6.25 (2.00-12.00) | 0.391 |

Abbreviations: Q1, first quartile; Q3, third quartile; p, p value; TN, triple negative, BC, breast cancer, HR, hormone receptors; CNS, central nervous system, MBC, metastatic breast cancer
