# Supplementary material for: Immune characterization of breast cancer metastases: prognostic implications
Source: Breast Cancer Res. 2018 Jun 22;20:62. doi: 10.1186/s13058-018-1003-1 (PMC6013851; doi:10.1186/s13058-018-1003-1)
Supplement: Supplementary file 3 — Table S3. CD8/FOXP3 ratio distribution according to tumor subtype and clinicopathological features. (DOCX 18 kb) [file 13058_2018_1003_MOESM3_ESM.docx]

**Table S3. CD8/FOXP3 ratio distribution according to tumor subtype and clinicopathological features.**

|  | **CD8/FOXP3 ratio median (Q1-Q3): all patients 3.20 (1.87-5.75)** | | | | | |
| --- | --- | --- | --- | --- | --- | --- |
|  | **Overall** | ***P*** | **TN cohort** | ***P*** | **HER2+ cohort** | ***P*** |
| **Tumor phenotype**  **TN**  **HER2+** | 3.00 (1.78-4.74)  3.35 (1.96-7.43) | 0.365 | -  - | - | -  - | - |
| **Age at BC diagnosis**  **≤50 years**  **>50 years** | 3.41 (2.05-5.86)  3.15 (1.72-5.05) | 0.354 | 3.96 (2.80-5.86)  2.26 (1.50-4.14) | 0.057 | 2.82 (1.99-5.86)  3.54 (1.77-7.50) | 0.634 |
| **HR status**  **Negative**  **Positive** | -  - | - | -  - | - | 2.37 (1.31-3.19)  3.91 (2.08-11.25) | 0.053 |
| **Site of biopsy**  **liver**  **skin**  **lung**  **CNS**  **Other** | 3.62 (1.72-11.25)  2.77 (1.50-4.14)  5.64 (1.57-21.03)  3.19 (1.99-4.43)  5.03 (2.55-7.50) | 0.343 | 1.52 (1.09-9.44)  2.80 (1.98-3.50)  2.62 (1.22-5.64)  4.74 (3.17-7.10)  5.86 (2.55-9.40) | 0.481 | 5.50 (2.64-11.25)  2.59 (1.40-4.55)  21.03 (10.15-40.03)  3.15 (1.99-3.19)  4.19 (1.77-7.50) | 0.089 |
| **Prebiopsy systemic treatment for MBC**  **No**  **Yes** | 3.48 (1.93-5.86)  2.73 (0.64-5.50) | 0.165 | 3.41 (1.91-5.05)  0.60 (0.57-3.20) | 0.062 | 3.62 (2.05-11.25)  2.82 (1.40-5.86) | 0.279 |

Abbreviations: Q1, first quartile; Q3, third quartile; p, p value; TN, triple negative, BC, breast cancer, HR, hormone receptors; CNS, central nervous system, MBC, metastatic breast cancer
