# Supplementary material for: Immune characterization of breast cancer metastases: prognostic implications
Source: Breast Cancer Res. 2018 Jun 22;20:62. doi: 10.1186/s13058-018-1003-1 (PMC6013851; doi:10.1186/s13058-018-1003-1)
Supplement: Supplementary file 4 — Table S4. PD-L1 levels distribution according to tumor subtype and clinicopathological features (DOCX 17 kb) [file 13058_2018_1003_MOESM4_ESM.docx]

**Table S4. PD-L1 levels distribution according to tumor subtype and clinicopathological features.**

|  | **PD-L1 median (Q1-Q3): all patients 5.00 (1.00-30.00)** | | | | | |
| --- | --- | --- | --- | --- | --- | --- |
|  | **Overall** | ***P*** | **TN cohort** | ***P*** | **HER2+ cohort** | ***P*** |
| **Tumor phenotype**  **TN**  **HER2+** | 10.00 (1.00-30.00)  5.00 (0.00-20.00) | 0.254 | -  - | - | -  - | - |
| **Age at BC diagnosis**  **≤50 years**  **>50 years** | 5.00 (0.00-30.00)  5.00 (1.00-30.00) | 0.848 | 12.50 (1.00-25.00)  10.00 (1.00-40.00) | 0.824 | 5.00 (0.00-30.00)  5.00 (1.00-10.00) | 0.803 |
| **HR status**  **Negative**  **Positive** | -  - | - | -  - | - | 12.50 (3.00-30.00)  5.00 (0.00-10.00) | 0.113 |
| **Site of biopsy**  **liver**  **skin**  **lung**  **CNS**  **Other** | 10.00 (3.00-30.00)  9.00 (0.00-20.00)  3.00 (0.50-40.00)  3.00 (1.00-30.00)  5.00 (0.00-5.00) | 0.781 | 10.00 (5.00-17.50)  15.00 (1.00-30.00)  3.00 (0.50-37.50)  20.00 (5.50-35.00)  5.00 (0.00-10.00) | 0.511 | 10.00 (3.00-30.00)  5.00 (0.00-10.00)  18.00 (0.50-40.00)  2.00 (0.00-3.00)  5.00 (0.00-5.00) | 0.694 |
| **Prebiopsy systemic treatment for MBC**  **No**  **Yes** | 10.00 (1.00-25.00)  3.00 (0.00-30.00) | 0.503 | 10.00 (1.00-30.00)  0.00 (0.00-60.00) | 0.483 | 5.00 (0.00-10.00)  3.00 (1.00-30.00) | 0.987 |

Abbreviations: Q1, first quartile; Q3, third quartile; p, p value; TN, triple negative, BC, breast cancer, HR, hormone receptors; CNS, central nervous system, MBC, metastatic breast cancer
