# Supplementary material for: Immune characterization of breast cancer metastases: prognostic implications
Source: Breast Cancer Res. 2018 Jun 22;20:62. doi: 10.1186/s13058-018-1003-1 (PMC6013851; doi:10.1186/s13058-018-1003-1)
Supplement: Supplementary file 5 — Figure S1. Overall survival for HER2+ patients according to CD8/FOXP3 ratio. (PPTX 80 kb) [file 13058_2018_1003_MOESM5_ESM.pptx]

## Slide 1
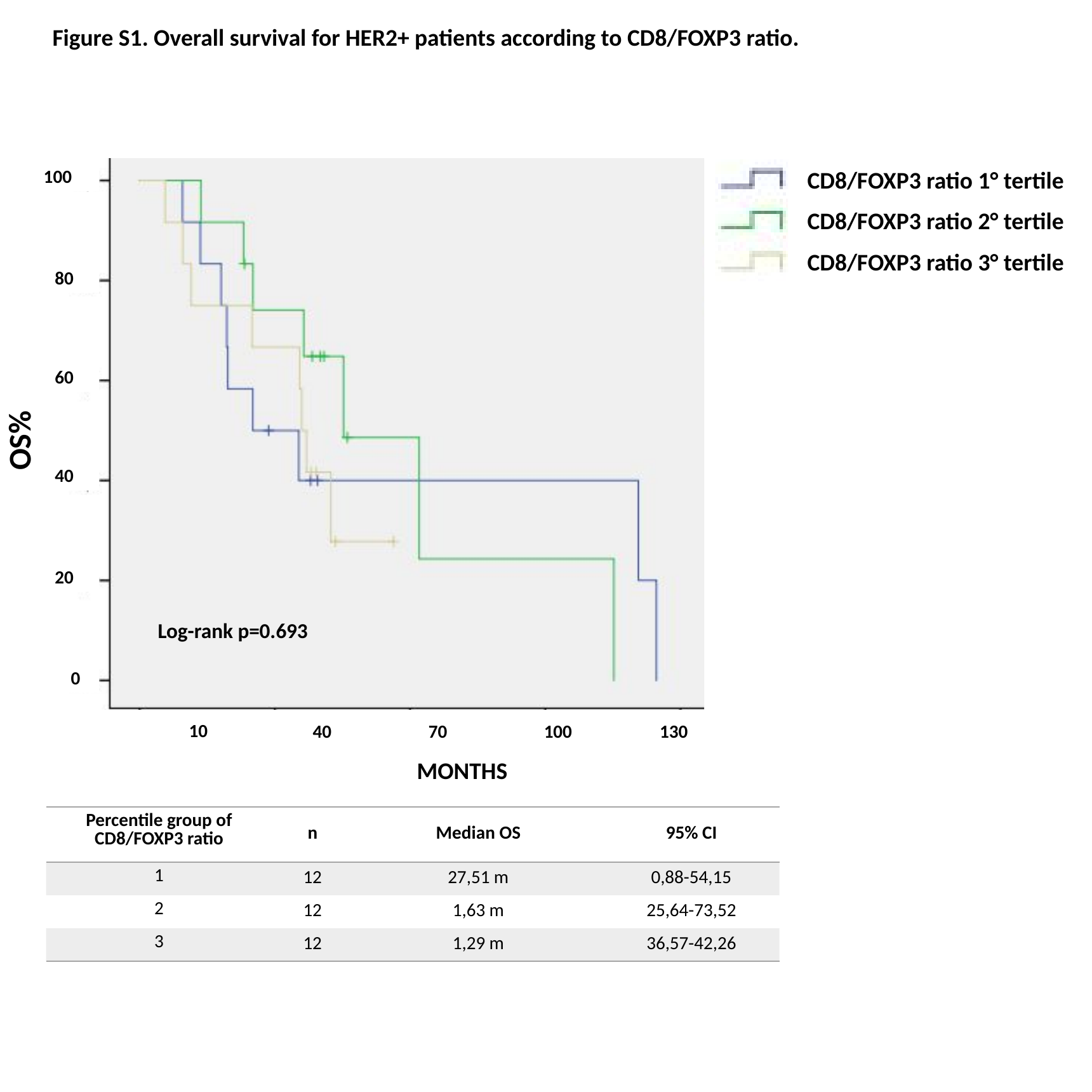

Figure S1. Overall survival for HER2+ patients according to CD8/FOXP3 ratio.
100
80
60
40
20
0
CD8/FOXP3 ratio 1° tertile
CD8/FOXP3 ratio 2° tertile
CD8/FOXP3 ratio 3° tertile
OS%
Log-rank p=0.693
10
40
70
100
130
MONTHS
| Percentile group of CD8/FOXP3 ratio | n | Median OS | 95% CI |
| --- | --- | --- | --- |
| 1 | 12 | 27,51 m | 0,88-54,15 |
| 2 | 12 | 1,63 m | 25,64-73,52 |
| 3 | 12 | 1,29 m | 36,57-42,26 |
