# Supplementary material for: Immune characterization of breast cancer metastases: prognostic implications
Source: Breast Cancer Res. 2018 Jun 22;20:62. doi: 10.1186/s13058-018-1003-1 (PMC6013851; doi:10.1186/s13058-018-1003-1)
Supplement: Supplementary file 6 — Figure S2. Overall survival according to PD-L1 expression in HER2+ patients (2A) and TN patients (2B). (PPTX 91 kb) [file 13058_2018_1003_MOESM6_ESM.pptx]

## Slide 1
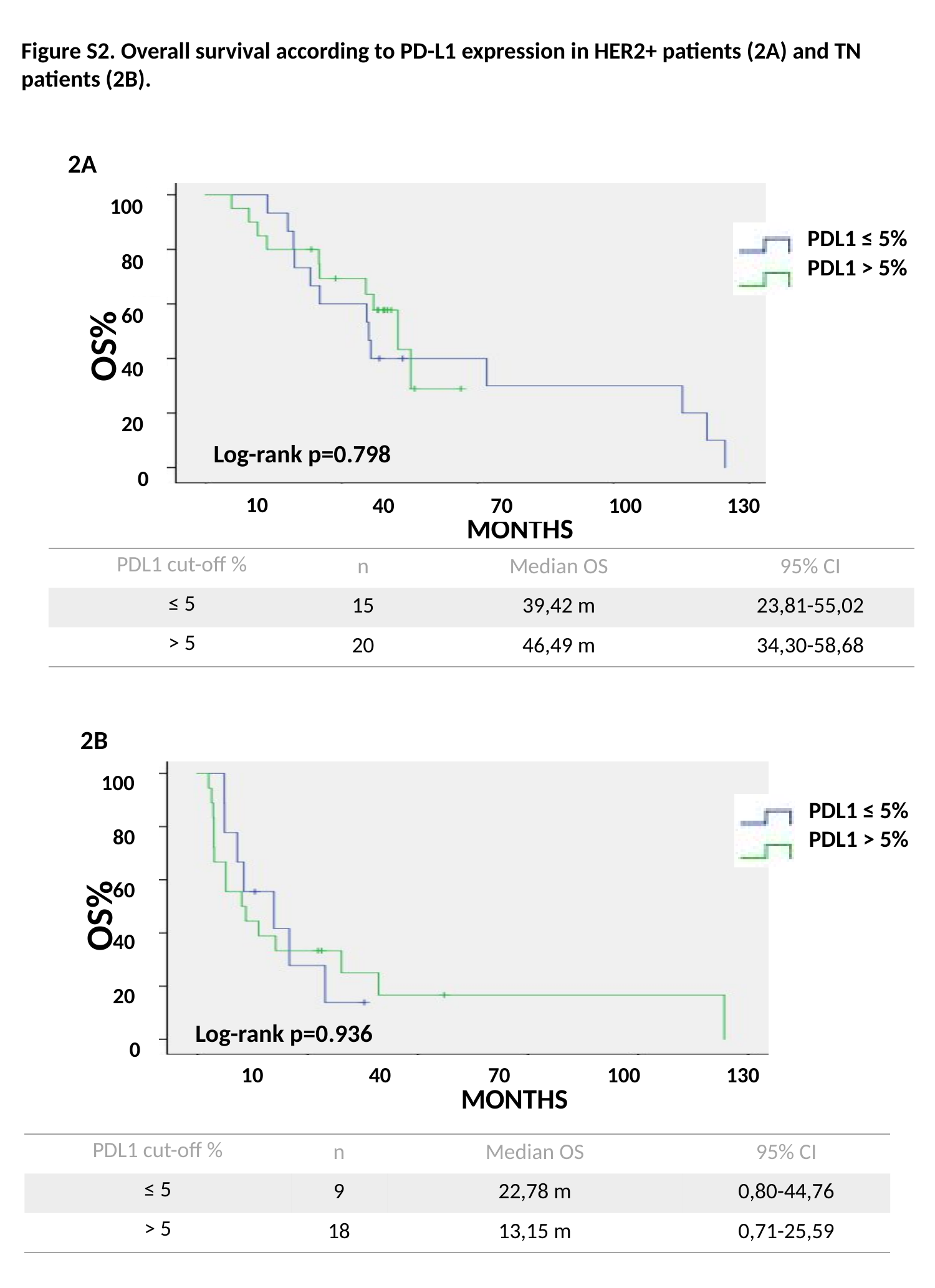

Figure S2. Overall survival according to PD-L1 expression in HER2+ patients (2A) and TN patients (2B).
2A
100
80
60
OS%
40
20
0
MONTHS
10
40
70
100
130
PDL1 ≤ 5%
PDL1 > 5%
Log-rank p=0.798
| PDL1 cut-off % | n | Median OS | 95% CI |
| --- | --- | --- | --- |
| ≤ 5 | 15 | 39,42 m | 23,81-55,02 |
| > 5 | 20 | 46,49 m | 34,30-58,68 |
2B
100
80
60
OS%
40
20
Log-rank p=0.936
0
10
40
70
100
130
MONTHS
PDL1 ≤ 5%
PDL1 > 5%
| PDL1 cut-off % | n | Median OS | 95% CI |
| --- | --- | --- | --- |
| ≤ 5 | 9 | 22,78 m | 0,80-44,76 |
| > 5 | 18 | 13,15 m | 0,71-25,59 |
